# Chondrogenic potential of manganese-loaded composite scaffold combined with chondrocytes for articular cartilage defect

**DOI:** 10.1007/s10856-022-06695-y

**Published:** 2022-10-11

**Authors:** Li Wei, Shuai Qin, Yulin Ye, Jiawei Hu, Danyang Luo, Yusi Li, Yiming Gao, Liting Jiang, Qi Zhou, Xianfei Xie, Ning Li

**Affiliations:** 1 Shanghai Institute of Traumatology and Orthopaedics, Ruijin Hospital, Shanghai Jiao Tong University School of Medicine, Shanghai, China; 2 Department of Critical Care Medicine, Ruijin Hospital, Shanghai Jiao Tong University School of Medicine, Shanghai, China; 3 Department of Stomatology, Ruijin Hospital, Shanghai Jiao Tong University School of Medicine; College of Stomatology, Shanghai Jiao Tong University, Shanghai, China; 4 Department of Orthopedics, Ruijin Hospital, Shanghai Jiao Tong University School of Medicine, Shanghai, China

## Abstract

Cartilage is an alymphatic, avascular and non-innervated tissue. Lack of potential regenerative capacity to reconstruct chondral defect has accelerated investigation and development of new strategy for cartilage repair. We prepared a manganese ion-incorporated natupolymer-based scaffold with chitosan-gelatin by freeze-drying procedure. The scaffold was characterized by Fourier transform infrared spectroscopy, thermogravimetric analysis, scanning electron microscopy, energy dispersive spectroscopy, compressive testing, and analysis of porosity and flexibility. Live/dead assay confirmed the good cytocompatibility of prepared scaffold on rat articular chondrocytes after 10 days and 4 weeks of culture. The manganese-loaded composite scaffold upregulated the expression of chondrogenic-related markers (Sox9, integrin, and Col II) in chondrocytes. Western blot analysis of proteins extracted from chondrocytes grown on scaffolds indicated the signaling pathways of p-Akt and p-ERK1/2 played a key role. Histological analysis following implantation of current composite scaffold loaded with chondrocytes into a rat articular cartilage defect model showed that the scaffolds promoted the formation of collagen II and cartilage repair. These findings suggested the potential of manganese-loaded scaffold to promote new cartilage formation and a promising strategy for articular cartilage engineering application.

## Introduction

Focal articular cartilage lesion is a common clinical problem of the knee joint, which leads to joint dysfunction, significant pain, swelling, and even disability [1]. Although surgical treatment is effective for many patients, current research focuses on preventing the progression of cartilage defect to osteoarthritis and regenerating natural hyaline cartilage. Due to the lack of blood vessels and lymphatic vessels, articular cartilage lacks the ability to repair spontaneously [2]. Therefore, cartilage repair remains a great challenge for clinicians and researchers. In recent decade, various scaffold biomaterials with potential three dimensional structure have been proven to be suitable for cartilage tissue engineering [3]. In the repair process, scaffolds generate a hydrated microenvironment that allows the exchange of nutrients and metabolic wastes, give the appropriate mechanical support from physiological loading, establish close cell-extracellular matrix contact to promote cell adhesion and retain the metabolic function of attached cells [4–6].

Several biomaterials of natural or synthetic origin have been used to fabricate the scaffolds [7]. Chitosan (CS) and gelatin as macromolecule nature-derived polymers have been widely applied for surgical, biomedical, and tissue engineering applications due to their muco-adhesiveness, biodegradability, and biocompatibility. Previous studies have shown that there is a structural similarity between the N-acetyl glucosamine group of chitosan and glycosaminoglycan found in cartilage extracellular matrix (ECM), which can create a more favorable chondrogenic microenvironment for cartilage tissue regeneration [6, 8]. Gelatin, a protein fragment, is another natural biomaterial that could promote cell adhesion, migration, proliferation and differentiation [9]. Therefore, chitosan-gelatin-based materials enhence the biological response and have been proposed as scaffold materials for regenerating cartilage, bone and skin [10–12]. Although chitosan or gelatin have satisfying bioproperties as graft material, their use is limited due to faster degradation rates, reduced bioactivites and mechanical properties [13]. These disadvantages could be overcome by adding other natural or synthetic polymers, or crosslinker molecules. Genipin (Gp), a naturally occurring crosslinking agent, is reported to be 10^4^ times less cytotoxic than glutaraldehyde [14]. It can improve the mechanical properties of scaffolds and affect their degradation rate directly [15]. It has been widely used as a crosslinker with various polymers, including chitosan and gelatin, which could be an attractive alternative for cartilage tissue engineering.

Recently, various biomaterials have been designed as a platform combined with seed cells for tissue engineering [16]. A biomaterial combined with mesenchymal stem cells for cartilage regeneration showed that the repair tissues had a better matrix staining and revealed biomechanical properties close to those of the normal cartilage [17]. Many clinical studies showed the effectiveness of scaffolds loaded with in vitro expanded chondrocytes for cartilage repair [18, 19]. It is an efficient way to perfuse high-density chondrocytes in porous scaffolds because of higher amounts of secretion of collagenous matrix and better cell differentiation behavior [20, 21].

In addition, metal ions incorporated in the scaffolds have been extensively investigated to enhance chondrocyte activity. Manganese (Mn) ion is an essential metal ion of vertebrate bones and teeth and has been shown to influence biological activities such as ECM remodeling and bone mineralization [22, 23]. Mn deficiency has been shown to cause a reduction in proteoglycan levels and qualitative changes in glycosylation, resulting in reduced osteogenesis and osteoclast activity [24, 25]. Previous studies revealed that Mn^2+^ incorporation in inorganic materials, including calcium phosphate, bioactive glass, is likely to improve their biological performance in terms of bioactivity and acceleration of bone mineralization [25, 26]. Therefore, it would be a good idea to explore the Mn^2+^ incorporation in polymer scaffolds for cartilage repair.

**Scheme 1:**
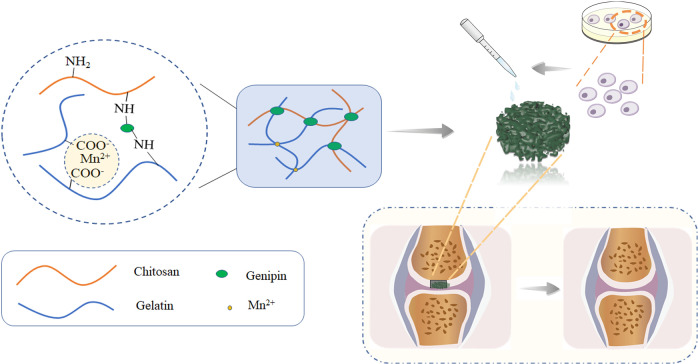
Schematic of the scaffold preparation and loading of cells onto a previously fabricated scaffold for treating rat articular defects

Herein, we developed and characterized a composite scaffold incorporated manganese ion loaded with chondrocytes for the treatment of cartilage defect. As shown in Scheme 1, Mn^2+^ incorporation porous scaffolds were prepared with Gp as crosslinker. Physical, morphological analyses and cellular compatibility were performed. Subsequently, we evaluated chondrogenic-related markers (Sox9, integrin, Col II) expression in chondrocytes cultured with composite scaffolds in vitro. Western blot assays for proteins extracted from chondrocytes grown on scaffolds indicated the role of p-Akt and p-ERK1/2 signaling pathways. In addition, the functionality of the Mn-incorporated scaffold was evaluated in vivo with a rat articular cartilage defect model.

## Materials and methods

### Materials

Geltain (Gel), chitosan (CS), Mn chloride was purchased from Macklin Biochemical Co. (Shanghai, China). Dulbecco’s Modified Eagle Medium (DMEM) and fetal bovine serum (FBS) was commercially obtained from HyClone (UT, USA). Other chemicals were obtained from Aladdin Industrial Co. Ltd. (Shanghai, China).

### Preparation of composite scaffolds

**Fig. 1:**
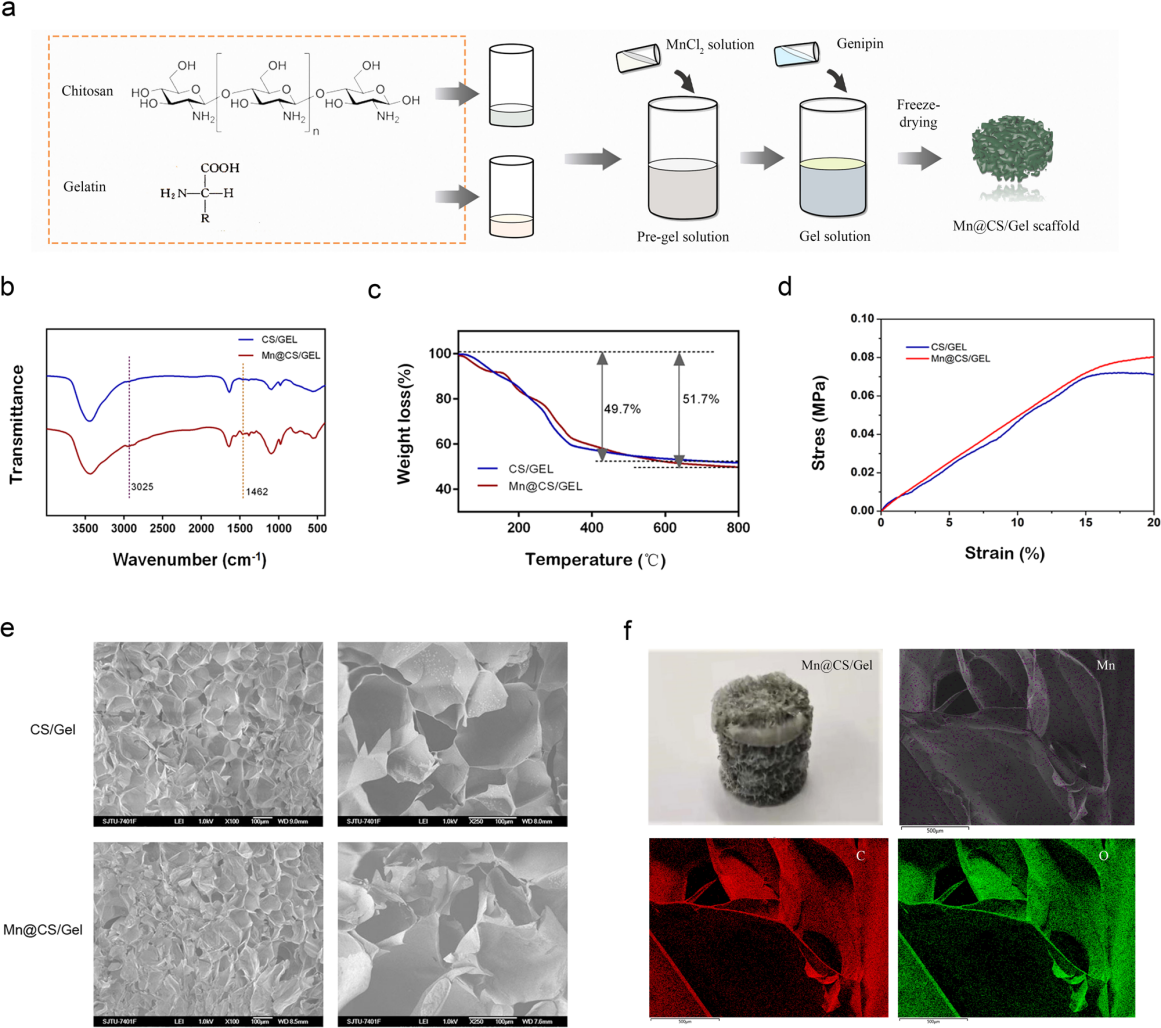
**a** Schematic illustration showing scaffold preparation processes; **b** FTIR spectra; **c** TGA curves; **d** compressive stress–strain curves of pristine scaffold and Mn-incorporated scaffold; **e** SEM images of the scaffold; **f** macroscopic image of the scaffold and EDS images of Mn, C, and O elements

Composite scaffolds were prepared as follows. In briefly, a given amount of CS (200 mg) was dissolved in 9 mL of 0.1 M Hydrochloric acid (HCl) solution to obtain a homogeneous solution. CS (2% w/v) and gelation (2% w/v) water solutions were mixed in a mass proportion of 60/40 (CS/Gel), then MnCl_2_ water solution was added to the mixture in proportion. An aqueous solution of Gp (1% w/v) was added dropwise to the prepared mixture solution. After that, the solutions were poured into 24 wells culture plate for the formation of hydrogel. The gels were basified in 0.1 M NaOH for 30 min prior to lyophilizing. The scaffolds without Mn^2+^ were prepared as control using the same process. The preparation processes for CS/Gel scaffold are shown in the Fig. 1a.

### Characterization of scaffolds

The characteristics of composite scaffolds were analyzed by FTIR; (Nicolet 5700, USA), thermogravimeter (TGA; PerkinElmer, USA), SEM (JEOL JSM-7401F, USA), and EDS (Sirion200, USA). The scaffolds were subjected to a universal testing machine (Instron 5569, Universal Testing system, USA) for compressive testing under a 1 mm/min moving condition. Then the porosity and pore size of the sponges were measured using ImageJ software according to the previous report [27].

### Isolation and culture of chondrocytes

**Fig. 2:**
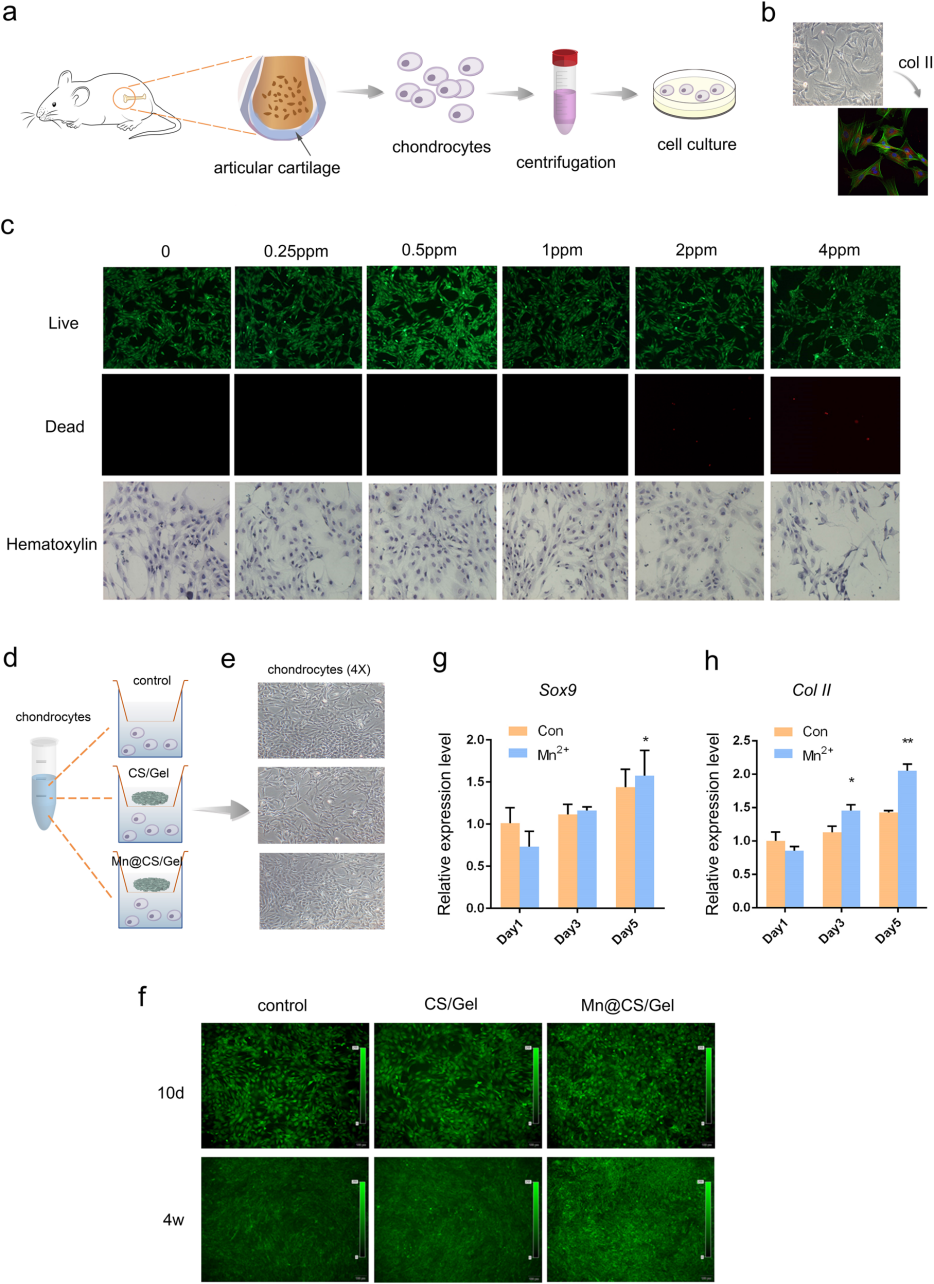
Biological assessment in vitro. **a** Schematic illustration showing chondrocyte isolation and culture processes; **b** confocal laser scanning microscopy of chondrocytes; **c** live/dead assay of chondrocytes on the scaffolds, live cells (green), dead cells (red) and HE stain at different concentration of Mn^2+^ after 3 days (bar = 200 μm); **d**, **e** schematic diagram and photographs of chondrocyte proliferation on day 3; **f** live cells (green) at 0.5 μg/mL Mn^2+^ concentration after 10 days and 4 weeks (bar = 100 μm); **g**, **h** expression of Sox9, Col II, and aggrecan genes by real-time PCR on days 1, 3, and 5 after incubation with pristine or Mn-incorporated scaffolds. (*p < 0.05 compared with the control group)

Articular cartilage from knee joints was obtained from newborn Sprague Dawley (SD) rats, then minced into small pieces of ~1 mm^3^. Subsequently, the articular cartilage fragments were digested with trypsin (Sigma-Aldrich, St. Louis, USA) for 5 min. Then, 0.2% type II collagenase (Sigma-Aldrich, St. Louis, USA) was added for 4 h on the water bath shaker at 37 °C, and the cell suspension was collected every 1 h. After termination of the digestion, the collected cell suspensions were mixed, centrifuged, and resuspended in DMEM supplemented with 10% (v/v) FBS (Gibco, Grand Island, NY, USA) and 1% (v/v) penicillin-streptomycin solution (Sigma-Aldrich, St. Louis, MO, USA) in a 5% CO2 incubator at 37 °C. The culture medium was replaced every 2 days, and the chondrocytes from passages 2–3 were harvested for subsequent experiments. The processes of chondrocytes isolation and culture are shown in Fig. 2a, and the marker of chondrocytes was observed using confocal laser scanning microscopy (Leica, Wetzlar, Germany).

### Cytotoxicity assay for the scaffold

The cytotoxicity of the scaffold was determined after 3 days and 4 weeks of cultivation. Calcein-AM/propidium iodide (PI) Live/dead double stain kit (Yeasen, Shanghai, China) was used following the manufacturer’s protocol. The scaffolds with the different concentrations of Mn2+ including 0, 0.25, 0.5, 1, 2, and 4 μg/mL were used to test the effects on cell viability. Chondrocytes (105 cells/well) were cultured with the above scaffolds on a 48-well plate for 3 days. Then, the live/dead double staining kit containing Calcein-AM and PI solutions stained live and dead cells, respectively. A 2 μM Calcein-AM and 4.5 μM PI staining solution was added to the samples and incubated in a 5% CO2 incubator at 37 °C for 30 min. Calcein fluorescence in living cells was excited at 490 nm by a fluorescent microscope (Carl Zeiss, Jena, Germany). Based on the above cell viability results, the scaffold with 0.5 μg/mL Mn2+ concentration was used to culture with chondrocytes on a 48-well plate for 4 weeks and assessed again. The chondrocytes proliferation was observed by an inverted phase contrast microscope (Olypusix70, IX-FLA, Japan) on day 3. The schematic diagram of chondrocytes proliferation in vitro is shown in Fig. 2d.

### Histological characterization and gene expression analyses

Coverslips were placed into wells of a 24-well plate for scaffolds and chondrocytes co-culture. After 3 days, coverslips were fixed in neutral buffered formalin (Sigma, 10% v/v). Immunohistochemical staining was performed for type II collagen (1:100, Abcam, USA). Immunofluorescence staining was investigated for integrin (1:100, Abcam, USA) (red) using a confocal laser scanning confocal microscope (Leica, Wetzlar, Germany). Cell cytoskeletons were stained with FITC-Phalloidin (Enzo Life Science Ltd., Exeter, UK) (green), and all cell nuclei were then counterstained with DAPI (Invitrogen) (blue). SOX9 and Col II gene expression levels of the chondrocytes were evaluated using RT–qPCR at 1, 3, and 5 days. The primer sequences used for the qPCR test were as follows: Sox9: TAGAACATTCACTGTGCCTTTG, CACTTACGGGCTGCTTGA; Col II: CCTCAAGGCAAAGTTGGTCCT, CTCCTGTCTCACCATACTTTG; actin: CATGTACGTTGCTATCCAGGC, CTCCTTAATGTCACGCACGAT. Total RNA was extracted using TRIZOL reagent (Invitrogen, USA). First-strand cDNA synthesis was performed using the PrimeScriptTM reverse transcriptase Reagent Kit (Takara Bio, Beijing, China) and then analyzed using SYBR Green (Takara Bio, Beijing, China) and an ABI7500 Sequence Detection system (Thermo Fisher Scientific, Waltham, USA). Actin was applied as an internal reference. All reactions were run in triplicate, and the relative expression of target genes was calculated using the 2 − ΔΔCt method.

### Western blot analysis

According to standard procedures, chondrocytes from each group were lysed in the buffer consisting of 200 µL RIPA lysis buffer (Beyotime, Shanghai, China), 2 µL PMSF (Sigma-Aldrich, St. Louis, USA), and 2 µL phosphatase inhibitor cocktail (Roche, Applied Science, Mannheim, Germany). The lysates were centrifuged at 14,000 *g* for 10 min at 4 °C, and the protein concentration was evaluated by Bradford protein assay (Bio-Rad Laboratories Inc., Hercules, CA, USA). The protein extracts were loaded and separated on 10% SDS-PAGE and then transferred to a polyvinylidene fluoride membrane (Millipore, Bedford, MA, USA). Transferred membranes were incubated in blocking solution with TBST buffer containing 5% w/v nonfat milk and incubated with primary antibodies as follows: anti-GAPDH antibody (1:1000, ab8245; Abcam), anti-AKT antibody (1:1000, #9272; Cell Signaling), phospho-AKT antibody (1:1000, #9271; Cell Signaling), p44/42 MAPK (Erk1/2) antibody (1:1000, #4696; Cell Signaling), and Phospho-p44/42 MAPK (Erk1/2) antibody (1:1000, #4370; Cell Signaling). It was incubated overnight in TBST (10 mM Tris/HCL, pH 7.5, 150 mM NaCl, 0.1% Tween-20) supplemented with 1% BSA at 4 °C. After hybridization with corresponding secondary antibodies from Cell Signaling, the membrane was visualized using an ECL western blotting detection system (ECL kits, #170-5060; Bio-Rad Laboratories Inc.). The results were digitized using a GE Image Quant LAS 4000 mini analyzer (GE, Marlborough, MA, USA). The relative abundance of four proteins was analyzed by obtaining the ratio of the normalized densitometric values. The experiments were performed in triplicate.

### In vivo experiment and assessment of cartilage repair

All procedures were approved by the Animal Care and Use Committee of Ruijin hospital. Eighteen male SD rats were used for the study. Under anesthesia by pentobarbital sodium (50 mg/kg), an osteochondral defect was created with a sterile electric drill (2 mm in diameter, 2 mm in depth) in the femoral trochlear groove of the hindlimb. Visible bleeding was observed to ensure that the defects reached the subchondral bone. Chondrocyte-loaded composite scaffolds or pristine scaffolds were randomly implanted into these defects of the femoral trochlea. The kneecap was restored, the knee capsule and muscular fascia tissue were sutured, and the skin incision was sutured. The rats were randomly divided into three groups: nontreated (control group, *n* = 6), scaffolds only (*n* = 6), and chondrocyte-loaded scaffold (*n* = 6). At 12 weeks postoperatively, six rats from each group were euthanized, and the samples were collected for histology and cellularity observation.

### Statistical analysis

Prism software was used for data analysis. All data were expressed as mean ± standard deviation and analyzed via one-way ANOVA followed by the Bonferroni test. The differences were considered statistically significant at *p* < 0.05.

## Results

### Characterization of scaffolds

As shown in Fig. 1b, the peaks of scaffolds at 1462 and 3025 cm^−1^ regions are ascribed respectively to the Mn-N and C-N stretching vibration, which confirmed the successful preparation of composite scaffolds. Thus, it was demonstrated that the prepared polymer scaffolds incorporated Mn^2+^ successfully.

Thermogravimetric analysis (TGA) of scaffolds was performed to analyze the influence of Mn^2+^ on scaffold structure. As shown in Fig. 1c, the weight loss of both scaffolds is approximate, demonstrating that adding manganese ions did not change the basic structural characteristics of the scaffolds. In addition, compared to the scaffold without manganese ions, adding 0.5 μg/mL Mn^2+^ concentration did not result in a significant increase in compressive stress (Fig. 1d).

Representative SEM images of scaffolds are shown in Fig. 1e. Irregular porosity was obtained. The pore size of the CS/Gel scaffolds was 100–300 μm with 68.1 ± 6.2% porosity, while the pore size of Mn-incorporated scaffolds was similar to the pristine scaffolds with 65.3 ± 5.7% porosity. Figure 1f exhibited the macroscopic image and the corresponding EDS mapping images of the scaffold. Mn, C, and O elements showed a uniform distribution in the scaffold.

### Cytocompatibility assay

We examined the effects of scaffolds with different Mn concentrations on the proliferation of chondrocytes. The images of chondrocytes after 3 days of contact with scaffolds incorporated with different concentrations of Mn^2+^ showed that the number of dead cells (red) increased significantly from that observed with 1 ppm concentration (Fig. 2c). Comparatively, the number of live cells in the 1–4 ppm group was slightly lower than that in the 0.5 ppm group. The findings of HE stain was similar to the live/dead results. Based on the above cell viability results, scaffolds with 0.5 ppm Mn2+ concentration were chosen for subsequent experiments. The chondrocytes proliferation ability was investigated by an inverted phase contrast microscope on day 3. As shown in Fig. 2e, the number of proliferating cells was not different in the three groups. Furthermore, a high predominance of live cells (green) over time (Fig. 2f) indicated a good cell viability response to the scaffolds for 4 weeks.

### Mn-incorporated scaffolds enhance chondrogenesis in vitro

Based on the good cell viability of scaffolds, composite scaffolds’ ability to promote chondrogenesis was tested by histological, gene expression analysis, and western blot assay.

SOX9 and Col II transcription levels were investigated in detail (Fig. 2g and h). The chondrocytes cultured on the Mn-incorporated scaffolds demonstrated higher Sox9 expression levels than that on pristine CS/Gel scaffolds on day 5 (*p* < 0.05). In addition, the expression levels of the gene increased over time. Notably, on day 1, it could also be observed that the cells cultured with Mn-incorporated scaffolds showed lower expression than pristine scaffolds but with no significance. Since Sox9 is a chondrogenic-related gene, it could be concluded that the burst release of Mn ion from the scaffolds greatly affects the chondrocytes’ metabolism.

**Fig. 3:**
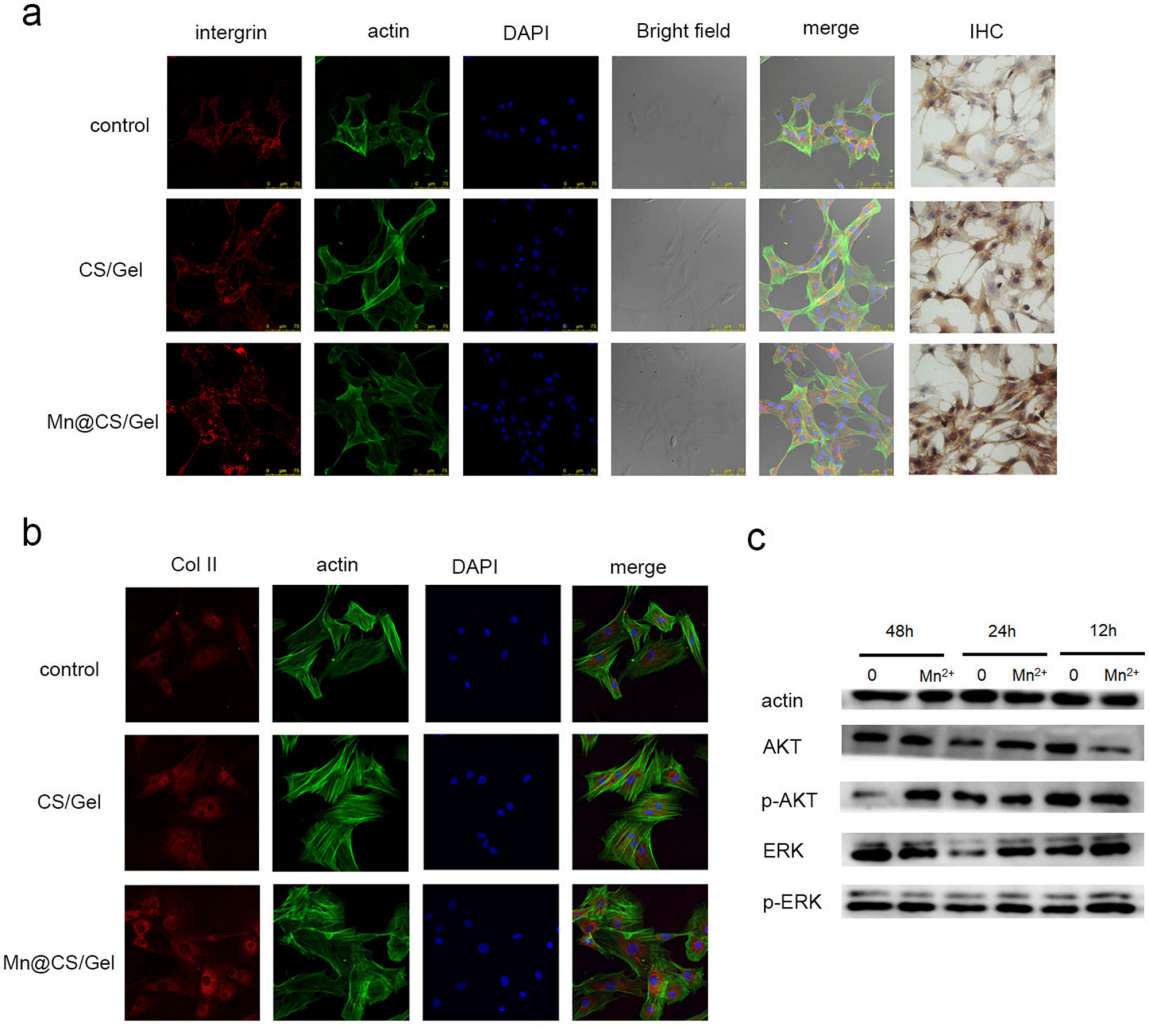
**a** Immunofluorescence and IHC staining images of integrin in chondrocytes co-cultured with the scaffold for 3 days; **b** representative immunofluorescence staining of Col II (red): actin cytoskeletons (green) and nuclei (blue) in chondrocytes co-cultured with the scaffold for 3 days; **c** western blot analysis of Mn ion-induced activation of the AKT/p-AKT and ERK/p-ERK pathways in chondrocytes

After 3 days of incubation with composite scaffolds, the protein expression of major chondrogenic markers (integrin and Col II) increased in the Mn-incorporated scaffold group in chondrocytes according to the immunofluorescence staining results (Fig. 3a and b).

Western blot assay was used to investigate whether 0.5 ppm concentration of Mn ion activated AKT or ERK signaling pathway. As expected, the western blotting results showed that the P-ERK1/2 and the p-AKT expression levels were activated at 24 and 48 h, respectively, in the chondrocytes incubated with the Mn-incorporated scaffolds compared with that of pristine scaffolds (Fig. 3c). Therefore, we speculate that the Mn ion may activate the AKT and ERK signaling pathways.

### In vivo osteochondral repair efficacy of scaffolds

**Fig. 4:**
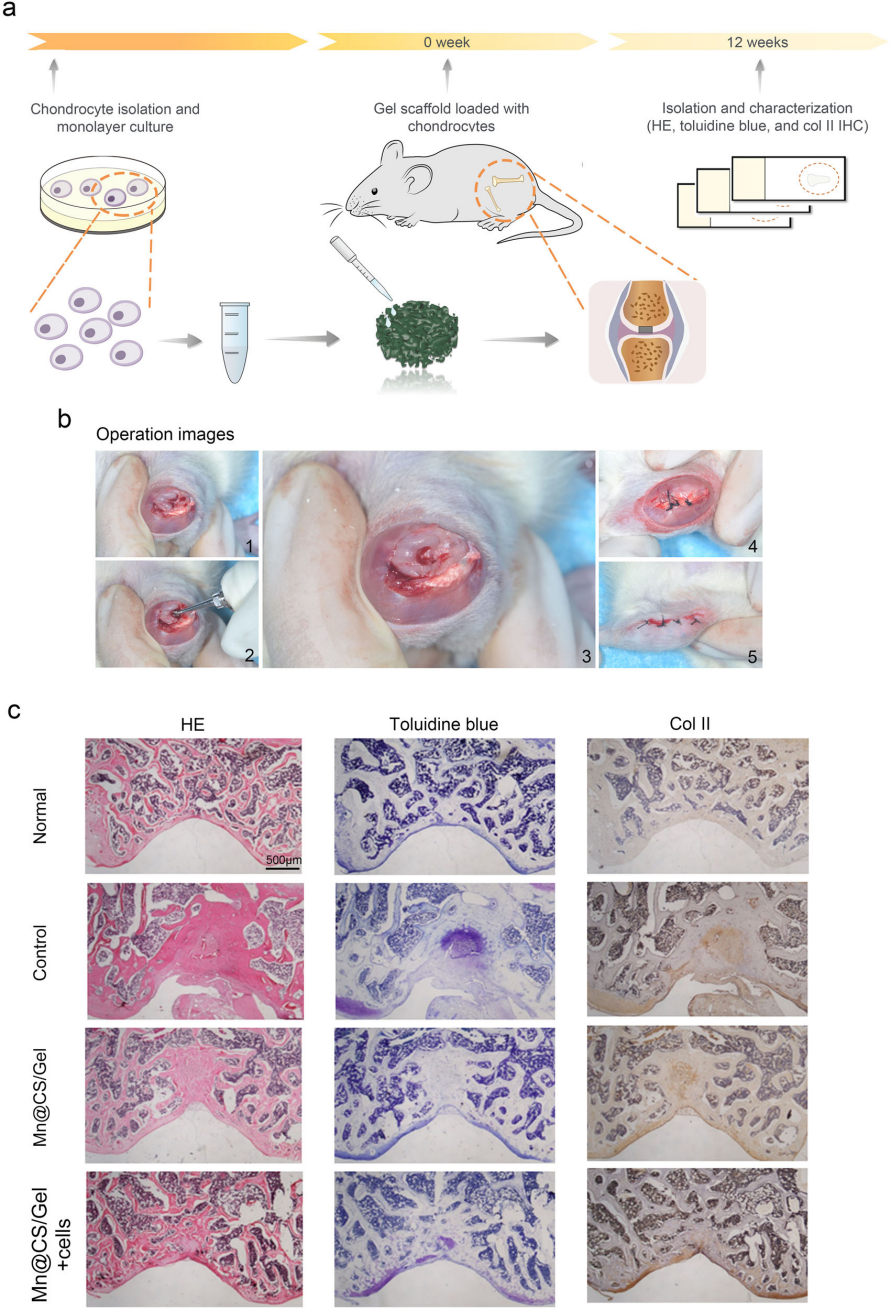
**a** Schematic diagram of osteochondral repair and histological analysis procedure in vivo; **b** The Operation of osteochondral defect in rats; **c** Histological analysis was performed by HE, Toluidine blue, and Col II staining

The operation procedure for an osteochondral defect in rats is shown in Fig. 4a, b. The defect with 2 mm diameter and 2 mm depth presented bad self-repair ability if scaffolds were not added. The histological analysis of the decalcified specimens was performed at 12 weeks post-operation. From Fig. 4c, more positive staining for collagen II was observed in Mn-incorporated scaffold combined with chondrocytes group compared with pristine scaffold through hematoxylin-eosin staining and toluidine blue staining. The above results indicated that the Mn-incorporated group had a better repair effect. Furthermore, more new osteochondral tissue was detected growing into the defects in the Mn-incorporated group than in the pristine scaffold group. No obvious osteochondral tissue was observed in the defect region in the blank control group.

## Discussion

In the present study, Fig. 1 shows that CS/gelatin scaffolds crosslinked genipin with or without Mn^2+^ presented similar pore sizes of 100–300 μm so that the seeded chondrocytes could adhere, spread, multiply, and secrete their matrixes onto the porous CS/gelatin scaffolds [28]. Many studies have shown that porous scaffolds have been effectively used in osteochondral tissue repair in defects [29]. However, the possible detrimental effects of scaffolds on mechanical properties increased with pore size. The genipin has been proven effective in enhancing the mechanical properties of the resulting materials [30]. In addition, as gelatin is negatively charged, the interaction between groups or ions could improve the properties of scaffolds for chondrocytes’ growth.

Although Mn ion was previously incorporated into bioceramics such as tricalcium phosphate under proper concentration and improved the bone mineralization rate [23], the appropriate Mn concentration within polymer remains uncertain. The live/dead results (Fig. 2b and c) showed that a scaffold doped with 0.5 ppm Mn concentration could keep the balance of toxicity and activity for chondrocytes. In a previous report, Fei et al. [31] examined the effects of different concentrations of Mn2+ on the proliferation of hBMSCs on the surface of the hydrogel. There was no statistically significant difference in the cell proliferation over the concentration range of 0–1 ppm. In contrast, the cell proliferation ability in the group of 1 ppm was slightly lower than that in the 0.5 ppm group. Therefore, it has been proved that the effect of Mn ion concentration is similar despite the different cell stems.

In vitro studies (Figs. 2 and 3) have shown that chondrocytes cultured with the Mn-incorporated scaffolds express elevated levels of chondrocyte markers like SOX9 and Col II compared to pristine scaffolds. Furthermore, it was found that Mn ion may induce the metabolic and proliferative capabilities of chondrocytes through activation of the signaling AKT and ERK signaling pathways (Fig. 3c), which suggests that they have a greater potential to restore articular cartilage.

The scaffold-based approach represents a fascinating treatment option for chondral lesions. There is an increasing number of published articles every year for various scaffolds of chondral defect repair. In addition, cells occupy a controversial role. Although using cell-free scaffolds showed good results and avoided cell manipulation and its regulatory obstacles, a scaffold/cell combination was the most investigated option [32]. Chondrocytes play a key role in cartilage development, homeostasis and tissue engineering [33]. Expansion and dedifferentiation of chondrocytes obtained from adult donors are accompanied by the loss of their chondrocyte phenotype and by a fundamental change of the chondrocyte gene expression profile with repression of typical chondrocyte marker genes like type II collagen [19]. As shown here for infant chondrocytes, chondrocyte re-differentiation and subsequent tissue maturation toward cartilage in vivo after transplantation accompanied by the timely degradation and resorption of the polymer-based scaffold has been a key factor. However, the lack of differentiation or maturation-promoting factors may result in cartilage tissue formation failure. The addition of growth factors, cytokines, and chemokines has shown better effects on chondrocytes, but the high price and rapid degradation limited the application widely [34–36]. Therefore, the bioactive manganese ions provide an alternative as stimuli factor.

As shown in Fig. 4, the investigation of rat cartilages has demonstrated that the scaffold/chondrocytes combination group for cartilage repair performs better than the cell-free scaffolds group. Through immune-staining for type II collagen, high expression profiles by the interaction between chondrocytes and Mn ion may promote infant chondrocytes’ potential.

## Conclusion

The present study demonstrated that a composite scaffold incorporated manganese ion and combined with chondrocytes perfuse was a good vehicle for cartilage repair. The scaffold with 0.5 ppm Mn^2+^ concentration could keep the balance of toxicity and activity for chondrocytes. Moreover, chondrocytes cultured with the Mn-incorporated scaffolds expressed elevated levels of chondrocyte markers like SOX9 and Col II compared to pristine scaffolds. The in vivo animal studies demonstrated that the scaffold/chondrocytes combination could significantly enhance new osteochondral tissue growing into the defects compared with the pristine scaffold. The results of this study suggested that this developed Mn-incorporated scaffold combined with infant chondrocytes may have the potential to restore articular cartilage.
